# Essays on Pathology and Therapeutics, Being the Substance of a Course of Lectures Delivered by Samuel Henry Dickson, M. D., Professor of the Institutes and Practice of Medicine in the Medical College of the State of South Carolina

**Published:** 1845-12

**Authors:** 


### BIBLIOGRAPHICAL NOTICES.

*Essays on Pathology and Therapeutics, being the substance of a Course of Lectures delivered* by SAMUEL HENRY DICKSON, M.D. Professor of the Institutes and Practice of Medicine in the Medical College of the State of South Carolina. 2 vols. oct. pp. 588, 651. Charleston, McCarter and Allen. 1845.

The size of this work, although but *the substance* of Dr. Dickson's Lectures, shows that the class to which his instruction was given enjoyed a pretty full view of that branch of medicine to which the learned and eloquent professor directed their attention. It is now welcomed by the profession, the intelligent members of which desire to see in one systematic work, the southern theory and practice of medicine. It is the more welcome because Professor Dickson, by position, educational advantages, and a constant intercourse with the profession in the South, enjoys unusual opportunities of making original observations, and of learning the common and accredited sentiment of southern physicians. It is, therefore, to us a subject of regret that we are restricted by the work for which we now write, to a mere notice; since such a survey and analysis as the book merits, would extend far beyond the limits of a monthly periodical. We shall, therefore, be excused by our readers for confining our observations to those parts of the work which possess the greatest originality, and on which the author has himself bestowed the most elaborate attention.

The work is divided into two parts. The first ten chapters treat of general subjects, such as causes of disease, Malaria, Contagion, Endemics, Epidemics, &c., while the great remainder of the book is devoted to special diseases or the " Practice of Physic." Every one who reads at all, must be aware of the great difficulty of treating well the subject of the first division of the work, since, on almost every question, there exist not only conflicting theories, but apparently contradictory facts. It is scarcely, therefore, matter of surprise that we should find in this part of the book much to receive our assent, and some things which seems to us rather paradoxical or illogical. The conclusiveness of the author's decisions in disputable matters, so necessary to the teacher, seem scarcely defensible in the writer; but is to be excused, since to avoid it would have demanded a re-formation of the whole work.

To the same circumstance we owe the loss of much very recent information, which could not be easily interwoven with the text of essays finished originally with care and skill. For example, Professor D. observes, in treating of malaria, that "Chemists have examined, with the most minute care, the constituents of the morbid atmospheres in which it has been supposed to abound." * * * "Neither by eudiometrical applications nor by the most perfect analysis can any additional ingredient of a gaseous nature, either in a state of mere mixture, or chemical combination, be detected in such air." The detection of sulphuretted products in marsh air by Daniel and Gardiner, and the discovery of azotized substances in marsh mists by M. Julia and others, are entirely overlooked.

The unquestionable position of the instructor has led to another occasional, though rare error, a careless quotation, or a misapprehension. Thus, for example, it is stated, vol. i. p. 58, that, "it is not during the rains that the malignant fevers of Africa arise"—whilst we know that the opposite is the fact, for the "Coast Fever" begins with the rains, and only ceases when the rainy season is over. Again: "We find the intensity or malignity of the diseases produced by it, (malaria), very directly proportioned to the degree and permanency of the heat of the locality affected. Thus in low latitudes, besides the terrible forms of pestilence just mentioned, (yellow fever and plague,) we have," &c. Again—"Like the plague, yellow fever belongs only to the heat of summer, and is extinguished by frost." Now almost every physician knows that yellow fever is most malignant in its most northern or coldest limits, and that the heat of June puts an end to the plague, so soon as the temperature transcends a given point.

We feel regret that so able a reasoner should not have noticed, in his felicitous way, the curious subject of the epidemic *cycle* of endemics. In this part of the American continent, malarial disease has been peculiarly extensive and severe, nearly every 20 years. Intermittents prevailed from 1801 to 1805—from 1820 to 1826—and in 1843 began to appear symptoms of the epidemic extensibility, which is severely felt in this 1845.

Despite such objections, we feel assured that no one can read the chapter on Malaria without both pleasure and profit.

The chapter on Contagion, learned and ingenious as it unquestionably is, will be esteemed by most readers of the present day as too bold in its general assertions. We cannot help thinking our author wrong in attributing to contagious matter the power of general and wide atmospheric diffusion, to which he appears to ascribe the epidemic extension of diseases ordinarily contagious. The experiments of rigid observers have demonstrated that even small pox is incapable of exerting its contagious powers beyond five or six feet, and that typhus fever has a still more limited sphere of infectious action. How then are we to believe that the measureless waste of air can be sometimes a mighty *fomes.* In the age of Sydenham, no one believed in the propagation of even small pox by contagion from man to man; now we are told that "all the febrile contagions, except vaccine, are capable of being thus (atmospherically) propagated—hence they may be styled diffusive or epidemic."

The chapter on "Seats of Diseases," abounds in knowledge and good sense, and shows that the author has, amidst fluctuating opinions and brilliant novelties, maintained a prudently eclectic position, a position now most necessary and yet almost impracticable, for the instructor. The very want of time to present every thing new and old to the learner, enforces an eclectic effort; but in making a selection, a bias is almost inevitable, and will be favourable or unfavourable to novelties, according to the warmth, or caution of the person who selects. Dr. Dickson, always a moderate humoralist, but by no means disposed to deny the influence of the solids in disease, is now, through the extravagance of recent humoralism, apparently an antagonist of his own system. To this condition must all prudent men come at last, in this age of sudden transitions and startling theories. Throughout this chapter we observe the habitual and salutary tendency to sift knowledge new and old, and to retain that which is profitable in both. Our author recommends statistical analysis, but admits that it "may be pushed too far, but it would be worse than idle to deny its very great utility." He also exposes the *undue* pretension of pathological anatomy, while he recommends its diligent cultivation. It will not teach us every thing, nor should it occupy us exclusively, but it is among the eminently useful means of knowledge.

We wish we could follow into more detail the course of our judicious author, in the general department of his work, since we believe few things would as much improve or gratify the readers of the Examiner; but time and space forbid us. It remains for us to notice briefly the learned Professor's views of remittent and yellow fevers, dengue, milk sickness, and one or two other complaints, on which his position, as well as his perspicacity and general authority, announce him as eminently a judge.

A hasty review of the theories of fever, brings our author to the admission that as yet too little is known of its efficient or proximate causes. He inclines to the belief that all fevers originate in some local irritation, but that we are far from being sure that the cause of fever affects exclusively the solids in the first instance. "Admitting the solids to be primarily affected, we do not know on which of them the first impression is made," "we do not know the nature of the primary impression, whether it be sedative or stimulating." "I repeat then my accordance in the ancient belief of the existence of a class of fevers properly contradistinguished as Idiopathic." (Vol. 1, p. 247.)

We have ever believed that conjectural medicine has done enormous mischief, by misdirecting inquiry, and misleading practice, and therefore decline the full expression of our dissent to the doctrine of our author, derived from arguments adduced by opposing theorists. It is enough to say that the observable or appreciable phenomena of fevers of any kind, are so distant from the unobserved initial processes, as to defy, at least now, the perspicacity of any philosopher. What is it that passes, in the frame of one who has received a variolous taint, antecedently to the access of a violent attack? And yet, for nine or ten, or even fifteen days, the poison is producing an unobserved suite of changes, no doubt momentous enough, if we knew how to detect it. So is it with other fevers, of all kinds, the causes of which, together with a prolonged chain of consequences, are latent, some of them at least, for very extended periods of time.

Since we know so little of the causes of fever, it would scarcely be profitable to follow the professor into his views of the sedative or stimulant nature of them.

The vexed question of the *jugulation* of fevers, is handled after the American manner, the "go-ahead and doubt not" method; so that Professor Dickson arranges himself with the mass of his countrymen, from Rush to Cooke, in the belief that fevers are susceptible of a sudden and total interruption by a judicious and vigorous practice. We profess to belong neither to the *expectants* nor the *heroics*. Fevers are sometimes interrupted by medicine, but usually the physician can only palliate, and conduct the disease to a safe termination. It is true that periodical fevers may be wonderfully suspended, and remittents now and then converted into intermittents, or conducted, in the remission, to a complete solution; but continued fevers present no good opening for the administration of the potent drug so felicitously applicable to the others, and such fevers are, so far as we know, never "jugulated."

As we ourselves incline to the rejection of critical days, we are pleased to find our author on the same side. In his mild and equable climate, rife with periodic diseases, the evidence for or against this ancient doctrine is readily obtained, and yet Professor Dickson declares that "to the mass of negative testimony on the subject, I must add my own, having failed entirely to satisfy myself of the influence of critical days, in the fevers of our climate."

An important remark is made on the greater frequency in South Carolina of the lesion of the gastro-intestinal surface in fevers,—our endemics being in this respect in singular contrast with English fevers, in which the cerebral and thoracic affections predominate largely.

We observe little that is original, but much that is judicious in the theory and practice in intermittents. The suggestion of the use of the cold bath in the hot stage, and of a combination of sulph. of quinia and sulphur in the intermission, is worthy of attention. Our author uses the cold bath not only in this but in most fevers, in which the strength of the patient does not forbid the exertion. To our minds, simple aspersion or affusion seems to answer every useful purpose, without being either so fatiguing or so hazardous as immersion.

Among the curiosities of the Museum of the College at Charleston, is a preparation of a *penis*, which became enlarged by congestion during the course of a prolonged intermittent, and assumed nearly that condition to which in similar cases the spleen is subjected. We regret that Professor Dickson did not state whether or not the congestion of the genital organ was at first paroxysmal, or suffered a gradual change.

Regret must be felt that our author does not touch the subject of congestive and algid intermittents, especially as we should like to have our Americanism gratified by a notice of the labours of Dr. Parry, of Indianapolis, and others of our countrymen, on this interesting subject.

The most important disease of the South, denominated by our author bilious remittent fever, is treated of, as it should be, copiously and minutely. Its causation is alleged to be solely an aërial miasm, the product of vegetable decomposition, a position by no means very defensible in the present state of knowledge on that subject. The books on marsh fevers abound in examples of decomposition without fevers, and of fevers without decomposition. Indeed, the profession at large is no longer generally favourable to the theory of *Lancisi*,—since it is known that the most malignant fevers prevail in the high and dry *Maremma*, while such diseases are almost unknown in the rich, wet, sunny plains of Brazil, and the hot luxuriant glades and stagnant marshes of Singapore. The letters of Mr. Peale and others, published by Prof. Dunglison in the pages of this journal, are extraordinary evidence of the uncertainty of our knowledge on this subject.

The remittent is the "*seasoning fever*," or acclimation of strangers, and the "*country fever*" of the inhabitants of Charleston. It is the scourge of all the low country of the South, a complete *taboo* to the residence of the white population upon rice and sugar and cotton plantations, during the summer season.

Among the symptoms of remittent fever, so well and so compactly described, we find two, which have been often esteemed almost peculiar to yellow fever; we mean the soft and swollen tongue, with its impressions received from the teeth; and an acute pain in the calves of the legs. We also notice, as somewhat peculiar, the remark that the Carolinian remittents, in the similar exacerbations of alternate, and the dissimilar ones of consecutive days, offer a very notable analogy to double tertians. They preserve throughout, in many cases, the particular complications of the kindred exacerbations; the head being most severely affected on the first, third and fifth days, the stomach or bowels, on the second, fourth and sixth. Even triple tertians are thus also closely followed.

The mortality of remittent fever in its usual form is much less than could be anticipated from the history of its symptoms. The deaths average in Charleston about 1 in 30; and for two years in the practice of our author, 1 in 52 nearly,—a proportional loss which must appear miraculous to those who denounce the mercurial treatment as criminally destructive. The character for truthfulness of the Professor, entitles him to implicit credit, both when he proclaims his method and asserts his success. The rate of mortality in congestive and dysenteric cases is higher, but these occur so rarely as not to influence much the result of general tables. In common cases, death arrives on, from the seventh to the eighteenth day; in those which pass into the typhoid state, the patients often linger for weeks, whilst those which become congestive or severely dysenteric, terminate in from three to ten or twelve days.

We do not remember to have seen elsewhere the judicious remark that anticipating exacerbations are unfavourable, and retarding ones favourable prognostics, whilst we are unprepared to admit that in any case of remittent fever "follicular ulceration may be found *throughout the whole extent of the bowels;* nay, we doubt its occurrence *even within its usual typhoid limits,* in the southern remittents.

We are not a little pleased with the treatment of our author, who combines courage with caution, who is not afraid to do enough, but is careful not to do too much. The knowing when to stop is as essential to the able physician, as to the finished orator. We are not however entirely satisfied with some points of practice laid down by the Professor. We think him a little behind the age in his cautionary monition respecting the use of quinia in remissions, and we cannot get entirely over our fear of the consequences of the cold dash and plunging bath so boldly urged in such cases.

In the typhoid state of remittents, Professor Dickson uses nitrate of silver beneficially he thinks. We have for several years urged upon the attention of our class the same remedy in the typhoid or entero-mesenteric fever, and believe that no other remedy exercises an equal control. We are therefore prepared to believe at once the favourable report of it here given. Remedies of this class seem to be attracting attention elsewhere; and Dobler, of Vienna, in a very recent paper declares, that in a certain typhoid epidemic, he found *alum* the only useful remedy, in any case, and at all stages.

Our limits, we are sorry to perceive, do not permit us to notice in farther detail, the excellent book of our author. We see ahead of us much to praise, something to blame, and a little to dispute. But as we must now take leave of Professor Dickson, we do it with less reluctance, because we hope to find at some other time a *caption* under which to present him and his useful labours to the readers of the Examiner. We cannot leave him, however, without thanks for the excellent arrangement, perspicuous language and manly propriety of his acceptable work,—for which, were we to designate it by a single epithet, we should choose the word *judicious*. M.

